# Developmental and activity-dependent modulation of coupling distance between release site and Ca^2+^ channel

**DOI:** 10.3389/fncel.2022.1037721

**Published:** 2022-10-26

**Authors:** Mitsuharu Midorikawa

**Affiliations:** Division of Neurophysiology, Department of Physiology, School of Medicine, Tokyo Women’s Medical University, Tokyo, Japan

**Keywords:** transmitter release, presynaptic terminal, coupling distance, development, long-term plasticity

## Abstract

Synapses are junctions between a presynaptic neuron and a postsynaptic cell specialized for fast and precise information transfer. The presynaptic terminal secretes neurotransmitters *via* exocytosis of synaptic vesicles. Exocytosis is a tightly regulated reaction that occurs within a millisecond of the arrival of an action potential. One crucial parameter in determining the characteristics of the transmitter release kinetics is the coupling distance between the release site and the Ca^2+^ channel. Still, the technical limitations have hindered detailed analysis from addressing how the coupling distance is regulated depending on the development or activity of the synapse. However, recent technical advances in electrophysiology and imaging are unveiling their different configurations in different conditions. Here, I will summarize developmental- and activity-dependent changes in the coupling distances revealed by recent studies.

## Introduction

The transmitter release from the presynaptic nerve terminal is triggered by an influx of Ca^2+^ from the voltage-gated calcium channels (VGCCs). Because the Ca^2+^ needs to diffuse from the channel pore to vesicular Ca^2+^ sensors, the coupling distance between VGCCs and Ca^2+^ sensors of releasable vesicles is a critical determinant of the release probability of the synaptic vesicles (Meinrenken et al., 2002; Wadel et al., 2007; Eggermann et al., 2011; Nakamura et al., 2015). The coupling distance is one of the key factors in producing functional heterogeneity of different synapses on the presynaptic side, which is also known to be modulated depending on development or synaptic activity (Ohana and Sakmann, 1998; Meinrenken et al., 2002; Fedchyshyn and Wang, 2005; Kochubey et al., 2009; Nakamura et al., 2015; Midorikawa and Miyata, 2021). It is one of the refinement processes at the synapse to establish an adaptive neural network.

The developmental change has been investigated mainly from unusually large presynaptic structures, such as the calyx of Held (Borst and Sakmann, 1996; Fedchyshyn and Wang, 2005; Nakamura et al., 2015) and the endbulb of Held (Oleskevich et al., 2004; Zhuang et al., 2017, 2020) at the auditory pathway. Technological advances have recently enabled recording from lemniscal fiber terminals (LFTs) at the sensory thalamus (Midorikawa and Miyata, 2021), providing another presynaptic model to digest developmental change in the coupling distance. The LFTs also provide an interesting model synapse to study selective strengthening and elimination (Midorikawa, 2022), but in this minireview, I will focus on the developmental change in the coupling distance. Because the calyx of Held terminal, endbulb of Held, and LFTs are all located in the middle of sensory pathways, they are good model synapses to investigate not only developmental but also experience-dependent modulation of the coupling distance.

The developmental/experience-dependent modulation is chronic changes that proceed over days. In addition to the chronic modulations, synapses are amenable to changing their function more acutely *via* acute intense activity, known as long-term synaptic potentiation and depression. Hippocampal mossy fiber bouton (hMFB) is a suitable model to investigate coupling distance before and after the long-term potentiation (LTP) since it is applicable for patch-clamp recording, and the LTP can be induced by a pharmacological manipulation (Weisskopf et al., 1994; Nicoll and Schmitz, 2005). Another form of acute plasticity is the so-called homeostatic plasticity of the *Drosophila* neuromuscular junction (NMJ) that occurs in response to postsynaptic impairments and leads to a compensatory increase of presynaptic transmitter release (Davis and Müller, 2015).

These studies have provided detailed information about acute and chronic modulation of coupling distance in different synapses. In this article, I would like to overview these various forms of coupling distance modulations.

## Manuscript formatting

### Headings

#### How voltage-gated calcium channel-releasable vesicle coupling distance affects the transmitter release kinetics

The coupling distance of VGCCs and release-ready synaptic vesicles is a key factor in determining the fidelity of the synaptic transmission. When an action potential (AP) arrives at the presynaptic terminal, Ca^2+^ influx through VGCCs triggers synaptic vesicle fusion and the release of transmitters stored in the synaptic vesicles. Therefore, the spatio-temporal profile of Ca^2+^ influx is the crucial determinant of the transmitter release, which is strongly affected by the AP waveform and the amount/distribution of functional Ca^2+^ channels. As for the AP waveform, broader AP is usually associated with a larger Ca^2+^ current because of the slower downstroke (Geiger and Jonas, 2000), resulting in a larger transmitter release (Augustine, 1990; Borst and Sakmann, 1999). Since the spatio-temporal profile of the Ca^2+^ is generally steep due to endogenous Ca^2+^ buffers (Nakamura et al., 2018), not only their numbers but also the distribution of VGCCs relative to the release-ready vesicles also plays a critical role in the efficacy of the transmitter release. The synaptic response can be described by multiplying a fixed number of the transmitter release site, mean release probability, and quantal response size (Takahashi, 2015; Sakaba, 2018). VGCCs-releasable vesicle coupling distance is a major determinant of the release probability among these parameters (Meinrenken et al., 2002; Eggermann et al., 2011; Nakamura et al., 2015).

Among central nervous system (CNS), some synapses have “loose” couplings (Rozov et al., 2001; Fedchyshyn and Wang, 2005; Vyleta and Jonas, 2014; Kawaguchi and Sakaba, 2017), while others have “tight” couplings (Bucurenciu et al., 2008; Eggermann et al., 2011; Schmidt et al., 2013; Kawaguchi and Sakaba, 2015). The different coupling distance results in a distinct pattern of transmitter release in response to incoming APs, which characterize various properties of CNS synapses. In general, “tight” coupling synapses have higher release probabilities than “loose” ones because they are exposed to a higher concentration of Ca^2+^. Because a large fraction of the readily releasable vesicles is depleted with the first AP, “tight” coupling synapses often demonstrate short-term depression, characterized by progressive weakening of transmitter release upon repetitive stimulations (Zucker and Regehr, 2002; Regehr, 2012). On the other hand, at some synapses with a low initial release probability due to “loose” coupling, repetitive stimuli can result in a progressive strengthening of synaptic responses, known as short-term facilitation (Zucker and Regehr, 2002; Abbott and Regehr, 2004).

It should be noted that besides AP waveform, amount of Ca^2+^ channels and the coupling distance of VGCCs and release-ready vesicles as shown above, there still are other factors that could affect synaptic vesicles’ release probability. A major alternative possible factor is heterogeneous Ca^2+^-sensitivity for transmitter release. Ca^2+^-sensors for the synaptic vesicle fusion synchronized to APs at the CNS are mediated mainly by Synaptotagmin-1, 2, or -9 (Südhof, 2013), but recent accumulating results indicate the critical role of synaptotagmin-7 on the asynchronous transmitter release (Bacaj et al., 2013; Luo and Südhof, 2017). Synaptotagmin-7 is also proposed to play a crucial role in short-term facilitation (Jackman et al., 2016; Turecek et al., 2017).

#### Developmental change of the coupling distance

**FIGURE 1:**
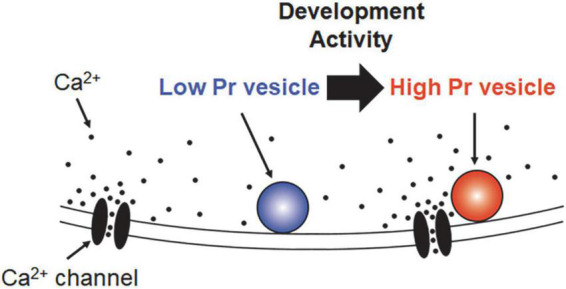
Schematic view of “loose” and “tight” coupling distance. The distance from the voltage-gated calcium channels (VGCCs) (or VGCC clusters) is different between “tight” (red) and “loose” (blue) coupled vesicles. The release probability of “tight” coupled vesicles is high because they are located close to the Ca^2+^ source, VGCCs, and hence exposed to a high concentration of Ca^2+^. On the other hand, the release probability of “loose” coupled vesicles is low because Ca^2+^ is diluted and buffered before reaching its location. The coupling distance could change from “loose” to “tight” depending on the development and/or activity.

**FIGURE 2:**
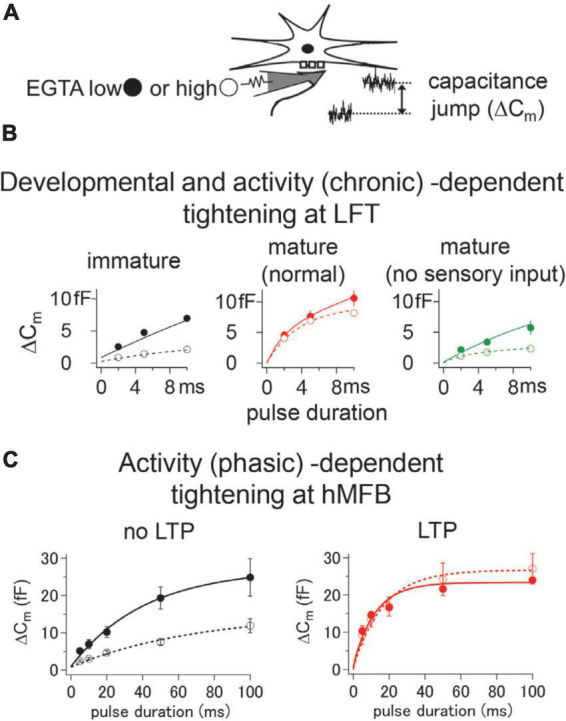
Developmental and chronic/phasic activity-dependent tightening of the coupling distance. **(A)** The coupling distance was examined by ethylene glycol tetraacetic acid (EGTA) sensitivity. Different amount of EGTA was injected into the presynaptic terminals through the patch-pipette, and the amount of transmitter release was detected by the capacitance measurement technique. **(B)** At lemniscal fiber terminal (LFT), the coupling distance showed developmental tightening, which can be seen by reduced sensitivity of ΔCm after maturation (compare black and red traces). The developmental tightening was impaired when the animal was grown up in a sensory-deprived condition (green traces). **(C)** At hippocampal mossy fiber bouton (hMFB), chemical-induced LTP caused tightening of the coupling distance, which was indicated by the reduction of the EGTA sensitivity in an LTP condition. All traces are adapted from Midorikawa and Sakaba (2017) and Midorikawa and Miyata (2021).

In addition to the intrinsic differences among different synapses, the coupling distance changes with development within the individual synapse. A number of studies have shown that the coupling is typically “loose” during early development, but the association becomes “tight” as synapses mature (Ohana and Sakmann, 1998; Meinrenken et al., 2002; Fedchyshyn and Wang, 2005; Figure 1). The coupling distance between VGCCs and releasable vesicles has been examined by investigating the sensitivity to a calcium chelator, e.g., EGTA (ethylene glycol tetraacetic acid), as a large amount of intracellular EGTA predominantly blocks exocytosis of loosely coupled vesicles (Adler et al., 1991; Borst and Sakmann, 1996). At the LFTs, located in the whisker-sensory pathway, the coupling distance is loose before the maturation (Midorikawa and Miyata, 2021). The coupling distance becomes tight at the beginning of the third postnatal week (∼P16), right after the timing when active whisking of rodents begins (Figure 2). Interestingly, the developmental tightening proceeds only at to-be-strengthened LFTs, but not at to-be-eliminated LFTs, despite synapsing onto the same postsynaptic neurons before the maturation (Midorikawa and Miyata, 2021). The developmental strengthening of the to-be-strengthened LFTs caused by the tightening of the coupling distance is postulated as one of the requirements to survive (Midorikawa, 2022). The study indicates that the developmental tightening of the coupling distance proceeds differently at distinct pathways.

The shortening of presynaptic AP wavelength is thought to represent a possible general mechanism underlying the developmental decrease of the release probability among different CNS synapses (Bolshakov and Siegelbaum, 1995; Pouzat and Hestrin, 1997; Taschenberger and Von Gersdorff, 2000; Midorikawa and Miyata, 2021). On the other hand, it has been reported that the amplitude of Ca^2+^ current enlarges to the mature level before the AP waveform maturation (shortening) (Taschenberger et al., 2002; Nakamura et al., 2015; Midorikawa and Miyata, 2021). At the calyx of Held, it is also shown that the number of VGCCs per cluster and the cluster area increase with development simultaneously (Nakamura et al., 2015). Developmental AP shortening decreases release probability while increasing the VGCCs, and/or the tighter coupling exhibit an antagonistic effect. Another comparison study between “weak” (low release probability) granule cell synapse and “strong” (high release probability) stellate cell synapse demonstrated that weaker granule cell synapse exhibited threefold more VGCCs than stronger stellate cell synapse, while the coupling distance was fivefold longer (Rebola et al., 2019). These studies indicate that the AP waveform, the number of VGCCs, and the coupling distance comprehensively regulate the developmental modulation of the transmitter release probability.

Because the coupling distance is crucial to determine the synaptic transmission property, extensive studies have been performed to investigate the regulatory mechanisms. For the coupling between VGCCs and release-ready synaptic vesicles, it has been shown that several vital proteins such as CAST/ELKS (Dong et al., 2018), neurexins (Missler et al., 2003; Luo et al., 2020), RIMs (Kiyonaka et al., 2007; Han et al., 2015), RIM-binding proteins (Acuna et al., 2015; Grauel et al., 2016; Butola et al., 2021), and (M)Unc13s (Böhme et al., 2016; Reddy-Alla et al., 2017; Kusch et al., 2018) have been proposed. Super molecular complexes consist of these proteins that differ from synapse to synapse, resulting in various synaptic transmission properties. Also, the expression and transportation of these proteins change with development, which could be postulated to underly a maturational tightening of the coupling distance. An interesting finding has been reported from studies of *Drosophila* NMJ, where Unc13A and -B are simultaneously present at mature presynaptic boutons, but those two isoforms arrive at different timing during synaptic development (Böhme et al., 2016). Unc13B, which mediates loose coupling, comes first and functions during the initial synapse assembly process. Unc13A, which mediates tight coupling, arrives later and mediates the vast majority of AP-evoked release at the mature synapse. Activity-dependent accumulations of the Unc13s are also observed (Böhme et al., 2016), which may shed light on the mechanism of activity-dependent coupling distance modulation in the future. Interestingly, (M)Unc13 proteins themselves might affect the coupling distance shift during maturation by recruiting Ca^2+^ channels to the active zone as shown at mammalian parallel fiber to Purkinje Cell synapse (Kusch et al., 2018), consistent with a direct interaction of VGCCs to Munc13s (Calloway et al., 2015). The presence of multiple isoforms of Munc13s and their loose redundancy on the transmitter release kinetics have also been reported at the calyx of Held synapse (Chen et al., 2013), at which developmental tightening of the coupling distance occurs (Nakamura et al., 2015).

#### Activity-dependent modulation of the coupling distance

The synapse formation can be roughly divided into three phases, initial axon targeting and contact with a target cell dendrite, organization of synapse structures to build the canonical synaptic construct shared by all synapses, and specification of synapse properties to confer unique features on each synapse (Südhof, 2017). Although the formation of long-distance axon tracts is restricted mainly to development and activity-independent, local branching of an axon is affected by neuronal activity (Uesaka et al., 2005, 2007). The following step, synapse formation, is a balancing process between formation and elimination. This phase is known to be activity-dependent and has been well studied in several brain regions (LeVay et al., 1980; Kano and Watanabe, 2019; Hooks and Chen, 2020; Midorikawa, 2022). The final step of synapse maturation is a specification of the synapse properties, which are largely affected by the neuronal activity to establish an adaptive neural network against the outer environment.

At the LFTs, continuous deprivation of sensory inputs from just before the onset of the active whisking prevents the developmental tightening of the coupling distance (Midorikawa and Miyata, 2021), which indicates that the process is experience-dependent (Figure 2). In this case, days of deprivation are required to impair the maturation process, suggesting that the tightening of the coupling distance is dependent on chronic activation of the pathway. On the other hand, the developmental shortening of the AP waveform is not affected by sensory deprivation at the LFTs (Midorikawa and Miyata, 2021). Therefore, at the LFTs developed without sensory experience, the shortened AP and the impaired coupling distance may result in unreliable synaptic transmission, leading to impaired sensory processing. In contrast to the unaffected AP waveform by sensory deprivation at LFTs, richer sensory inputs could induce shortenings of the AP waveform at other synapses. AP waveforms of specific neurons in the amygdala, cochlear nucleus, and cerebellum have been shown to be shortened by fear extinction, noise exposure, and enriched environment, respectively (Senn et al., 2014; Ngodup et al., 2015; Eshra et al., 2019). In these cases, tightening of the coupling distance or/and enlargement of the Ca^2+^ current can be postulated as compensatory mechanisms to maintain or facilitate reliable synaptic transmission.

Unlike LFTs, the kinetics of the transmitter release is not affected by the sensory deprivation at the calyx of Held synapse (Oleskevich et al., 2004), suggesting that developmental tightening of the coupling distance is not dependent on the hearing experience at this synapse. Interestingly at the endbulb synapse, which is located at the hearing pathway one synapse before the calyx of Held synapse, release probability is increased *via* hearing deprivation during the development (Oleskevich et al., 2004). Here, the increase in the release probability is not due to the tightening of the coupling distance but to the enhancement of the Ca^2+^ current (Zhuang et al., 2020).

These studies indicate that the activity-dependent developmental modulation of the release probability could work either in a Hebbian plasticity mode (inactive synapse becomes weak, i.e., LFT) or homeostatic plasticity mode (inactive synapse becomes strong, i.e., endbulb of Held).

Besides the chronic activity-dependent modulation of the coupling distance and/or transmitter release kinetics, as shown above, more phasic activity also could change the kinetics of transmitter release by the coupling distance modulation. One such kind of presynaptic plasticity is the homeostatic plasticity of the *Drosophila* NMJ, which induces a compensatory increase of presynaptic transmitter release in response to postsynaptic receptor blockade (Davis and Müller, 2015). Here, it has been shown that the enhanced transmitter release following the homeostatic plasticity had increased sensitivity to Ca^2+^ chelator EGTA, indicating that more loosely coupled vesicles are triggered for release under such a condition (Wentzel et al., 2018). Another presynaptic plasticity induced by phasic activity is the LTP of the neuron implicated in learning and memory. Although early studies of long-term synaptic plasticity described a potentiation of postsynaptic signal transduction mechanisms, it is now apparent that there is a vast array of presynaptic mechanisms for LTP (Yang and Calakos, 2013). Arguably best-characterized locus of the presynaptic LTP is the hippocampal mossy fiber synapse, where presynaptic plasticity was first found (Zalutsky and Nicoll, 1990). Several lines of studies, including quantal analysis of EPSCs (Malinow and Tsien, 1990), paired-pulse ratio analysis (Zalutsky and Nicoll, 1990), and monitoring progressive irreversible blockade of NMDA receptor-mediated EPSCs (Weisskopf and Nicoll, 1995) indicate that the LTP of this synapse is caused by an increase in release probability. Here, LTP depends on elevated cAMP levels, protein kinase A activation, and the phosphorylation of presynaptic substrates (Huang et al., 1994; Weisskopf et al., 1994). Because of its large size, hMFB is one of the few presynaptic structures amenable to direct patch-clamp recording (Geiger and Jonas, 2000; Hallermann et al., 2003; Vyleta and Jonas, 2014; Midorikawa and Sakaba, 2017). Direct infusion of cAMP into hMFBs through the patch pipette increased the release probability, accompanied by a reduced EGTA sensitivity, suggesting a tightening of the coupling distance (Midorikawa and Sakaba, 2017; Figure 2). Further study using super-resolution microscopy revealed that the cAMP-induced tighter coupling was caused by the physical expansion of VGCC clusters (Fukaya et al., 2021). It has been shown that the mobility of Ca^2+^ channels is high with presynaptic terminals (Schneider et al., 2015), and active zones recruit Ca^2+^ channels *via* active zone scaffolding proteins such as RIM, RIM-binding proteins, and Neurexins (Kaeser et al., 2011; Liu et al., 2011; Acuna et al., 2015; Han et al., 2015; Luo et al., 2020). Tightening of the coupling distance accompanied with LTP at the hMFB may be caused by the recruitment of VGCCs to active zones by scaffolding factors (Lübbert et al., 2019; Held et al., 2020).

#### Conclusion and future direction

As overviewed here, the coupling distance between VGCCs and release-ready vesicles is one of the key regulatory principles for determining synaptic behavior. It varies remarkably among different types of synapses, and even within the individual synapse, the coupling distance can be modulated by the chronic (e.g., development, experience-dependent) or phasic (e.g., LTP) activity of the synapse. Recently, the coupling distance has been estimated based on the kinetics of transmitter release measured *via* electrophysiology, electron microscopy, and model simulation (Budisantoso et al., 2012; Vyleta and Jonas, 2014; Nakamura et al., 2015; Böhme et al., 2016; Rebola et al., 2019). The studies have suggested various coupling distances and distribution patterns of VGCCs and release sites at different synapses under different conditions. Furthermore, the recent evolution of super-resolution microscopy enables us to visualize VGCCs and release site markers, such as Munc13 (Sakamoto et al., 2018), simultaneously to directly measure the coupling distance (Fukaya et al., 2021). The technical advances are shedding light on variable coupling distances among different synapses and their phasic and chronic plasticity.

It is interesting to ask what is shared and what is different between developmental and activity-dependent modulation of coupling distance. The initial triggering signal might be different since basal synaptogenesis mechanisms, and functional maturation of at least a subset of synapses are activity-independent (Verhage et al., 2000; Sando et al., 2017; Sigler et al., 2017). However, the signaling cascade might be converged and shared to some degree.

The ideal experiment will be to visualize the exocytosis of a single synaptic vesicle with Ca^2+^ influx from VGCCs. Simultaneous measurement of exocytosis of individual synaptic vesicle together with Ca^2+^ influx (Midorikawa et al., 2007; Midorikawa and Sakaba, 2015) or an active zone marker (Midorikawa et al., 2007; Zenisek, 2008; Joselevitch and Zenisek, 2020) has been performed using total internal reflection fluorescence microscopy, but the diffraction-limited spatial resolution (∼ 200 nm in) is far from sufficient to argue the coupling distance, which is typically in the range of tens to a hundred nanometers. The rapid expansion of live imaging techniques equipped with spatial resolution above the diffraction limit and fast temporal resolution will identify the coupling distance of various synapses in a different state, which will be fruitful for our understanding of the adaptive refinement of the presynaptic transmitter release machinery in the future.

## Author contributions

MM wrote the manuscript and prepared the figures.
